# *XPG* rs2296147 T>C polymorphism predicted clinical outcome in colorectal cancer

**DOI:** 10.18632/oncotarget.7352

**Published:** 2016-02-12

**Authors:** Fang Wang, Shao-Dan Zhang, Hong-Mei Xu, Jin-Hong Zhu, Rui-Xi Hua, Wen-Qiong Xue, Xi-Zhao Li, Tong-Min Wang, Jing He, Wei-Hua Jia

**Affiliations:** ^1^ Sun Yat-Sen University Cancer Center, State Key Laboratory of Oncology in South China, Department of Experimental Research, Collaborative Innovation Center for Cancer Medicine, Guangzhou 510060, Guangdong, China; ^2^ Department of Pediatric Surgery, Guangzhou Women and Children's Medical Center, Guangzhou Medical University, Guangzhou 510623, Guangdong, China; ^3^ Reproductive Medical Center, Department of Obstetrics and Gynecology, Sun Yat-Sen Memorial Hospital, Guangzhou 510120, Guangdong, China; ^4^ Molecular Epidemiology Laboratory and Department of Laboratory Medicine, Harbin Medical University Cancer Hospital, Harbin 150081, Heilongjiang, China; ^5^ Department of Oncology, The First Affiliated Hospital of Sun Yat-Sen University, Guangzhou 510080, Guangdong, China

**Keywords:** colorectal cancer, xeroderma pigmentosum group G, single nucleotide polymorphism, progression-free survival, overall survival

## Abstract

Xeroderma pigmentosum group G (XPG), one of key components of nucleotide excision repair pathway (NER), is involved in excision repair of UV-induced DNA damage. Single nucleotide polymorphisms (SNPs) in the *XPG* gene have been reported to associate with the clinical outcome of various cancer patients. We aimed to assess the impact of four potentially functional SNPs (rs2094258 C>T, rs2296147 T>C, rs751402 G>A, and rs873601 G>A) in the *XPG* gene on prognosis in colorectal cancer (CRC) patients. A total of 1901 patients diagnosed with pathologically confirmed CRC were genotyped for four *XPG* polymorphisms. Cox proportional hazards model analysis controlled for several confounding factors was conducted to compute hazard ratios (HRs) and 95% confidence intervals (CIs). Of the four included SNPs, only rs2296147 was shown to significantly affect progression-free survival (PFS) in CRC. Patients carrying rs2296147 CT/TT genotype had a significantly shorter median 10 years PFS than those carrying CC genotype (88.5 months vs. 118.1 months), and an increased progression risk were observed with rs2296147 (HR = 1.324, 95% CI = 1.046–1.667). Moreover, none of the four SNPs were associated with overall survival. In conclusion, our study showed that *XPG* rs2296147 CT/TT variants conferred significant survival disadvantage in CRC patients in term of PFS. *XPG* rs2296147 polymorphism could be predictive of unfavorable prognosis of CRC patients.

## INTRODUCTION

Colorectal cancer (CRC) is the third most common cancer and the fourth leading cause of cancer-related death in the world (http://globocan.iarc.fr/Pages/fact_sheets_cancer.aspx). Incidence of CRC dramatically varies from region to region. It ranks fifth in the commonly diagnosed malignancies in China, with 253, 427 new cases diagnosed and 139, 416 cancer deaths in 2012 (http://globocan.iarc.fr/Pages/fact_sheets_population.aspx).

It ranks the third in the commonly diagnosed malignancies in males and the second in females, with 1.4 million new cases diagnosed and 693, 900 cancer deaths in 2012 [1]. Therefore, it remains a major public health problem in China. Physicians make treatment plan depending on the clinical stage, performance status, and molecular characteristic of the tumor. Generally, surgery is used to treat the early stage of CRC, while the combination of 5-fluorouracil (5-FU), irinotecan, and oxaliplatin (FOLFOXIRI) is administrated to late-stage patients as the standard first-line chemotherapy to improve the prognosis [2]. The prognosis of colorectal cancer has been gradually improved over the past decades, with a 5-year relative survival of 65% and less than 50% in high- and low-income countries, respectively [3].

CRC is a complex disease, and both environmental and genetic factors contribute to oncogenesis. Diet (e.g., red meat) [4], smoking [5], drinking [6] and obesity [7] are well-known risk factors for CRC, although the underlying mechanisms remain clarified. Apart from those environmental factors, numerous evidence suggests that DNA repair systems also play an important role in modifying the risk of CRC [8–10]. For example, SNPs in *XPD* have an effect on the prognosis of CRC patients who were treated with oxaliplatin and 5-fluorouracil. Comparing to *XPD* 751Lys/Lys genotype, patients carrying Lys/Gln genotypes had more prone to chemotherapy failure and patients carrying ≥ 1 Gln had shorter median disease progression [8]. DNA repair systems include nucleotide excision repair (NER), base excision repair (BER), mis-match repair (MMR) and double-strand break repair (DSBR) pathways [11]. Of them, NER is responsible for repairing ultraviolet light (UV)-DNA damage, bulky DNA adducts (thymine dimers and 6, 4-photoproducts). NER functions properly by orchestrating different functional proteins involved in this pathway for the recognition of DNA lesion, incision, repair, and ligation [12]. Xeroderma pigmentosum group G (XPG) is one of the critical proteins in the NER pathway, encoded by excision repair cross-complementation group 5 (ERCC5) [12]. Accumulating studies have shown that the functional single nucleotide polymorphisms (SNPs) in the *XPG* gene may modify DNA repair capacity, and consequently increase the instability of genome. Additionally, such SNPs may also influence the ability to repair DNA damage caused by chemotherapeutic drugs, which enhances chemotherapeutic sensitivity and improve prognosis of CRC patients [13–15]. *XPG* is located on chromosome 13q33, containing 15 exons. Its protein product, an 1186-amino acid protein, is a structure-specific endonuclease responsible for the 3′ incision of DNA damage during NER, which has preference for the binding site of the single strand and the double strand of the degeneration bubble [16].

It has been reported that the SNPs in the *XPG* gene play a vital role in the outcomes of various cancers, including gastric carcinoma [17, 18], non-small cell lung cancer (NSCLC) [19], breast cancer [20]. However, the association between CRC and *XPG* polymorphisms remain controversial [21]. Therefore, the aim of our study was to assess the association of four potentially functional SNPs of *XPG* (rs2094258 C>T, rs2296147 T>C, rs751402 G>A, and rs873601 G>A) with over-all survival (OS) and progression-free survival (PFS) of CRC in 1901 Chinese CRC patients.

## RESULTS

### Patient characteristics and clinicopathological cutcomes

The demographic and clinicopathological characteristics of 1901 patients were shown in Table 1. Patients aged between 13 to 91 years, with a median age of 57.05 years. Body mass index (BMI) ranged from 13.19–41.36 (median: 22.38) and 60.5% of the patients were males. Most of patients did not have unhealthy lifestyle, such as smoking and drinking, and most of the patients with later Dukes stages. Patients with rectum and colon cancer accounted for 54.2% and 45.8% of CRC, respectively. After surgery and chemotherapy, 96.8% patients showed good response and about 1/3 patients later underwent recurrence or metastasis.

**Table 1:** Demographic and clinical data of patients

Variables	Number	Range/Percentage (%)
Age (years) 58.00	916 (> 58.00)	13–91 (range)
BMI (weight/hight^2^) 22.23	840 (> 22.23)	13.19–41.36 (range)
Patients with PFS	1516	79.7
Patients without PFS	385	20.3
Sex		
Male	1150	60.5
Female	751	39.5
Smoking status		
Never	1381	73.2
Ever	505	26.8
Drinking status		
Never	1599	85.0
Ever	282	15.0
Dukes stage		
A	207	10.9
B	629	33.2
C	608	32.1
D	449	23.7
Tumor site		
Rectum	977	54.2
Colon	824	45.8
Therapeutic response		
Better	1716	96.8
Worse	57	3.2
Recurrence/Metastasis status		
No	1197	63.0
Yes	704	37.0
Therapeutic methods		
Surgery	1001	52.7
Chemotherapy	708	37.2
Radiotherapy	59	3.1

BMI, body mass index.

### Association between *XPG* SNPs and prognosis of CRC

**Figure 1:**
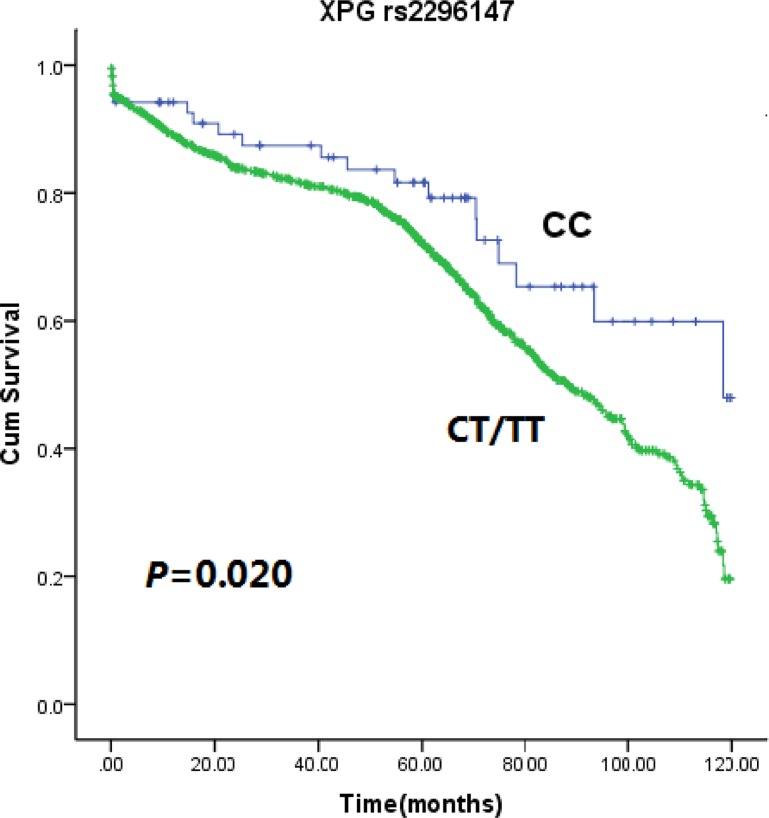
Kaplan-Meier estimates of 10 years PFS with *XPG* rs2296147 CT/TT and CC genotypes in CRC patients

**Figure 2:**
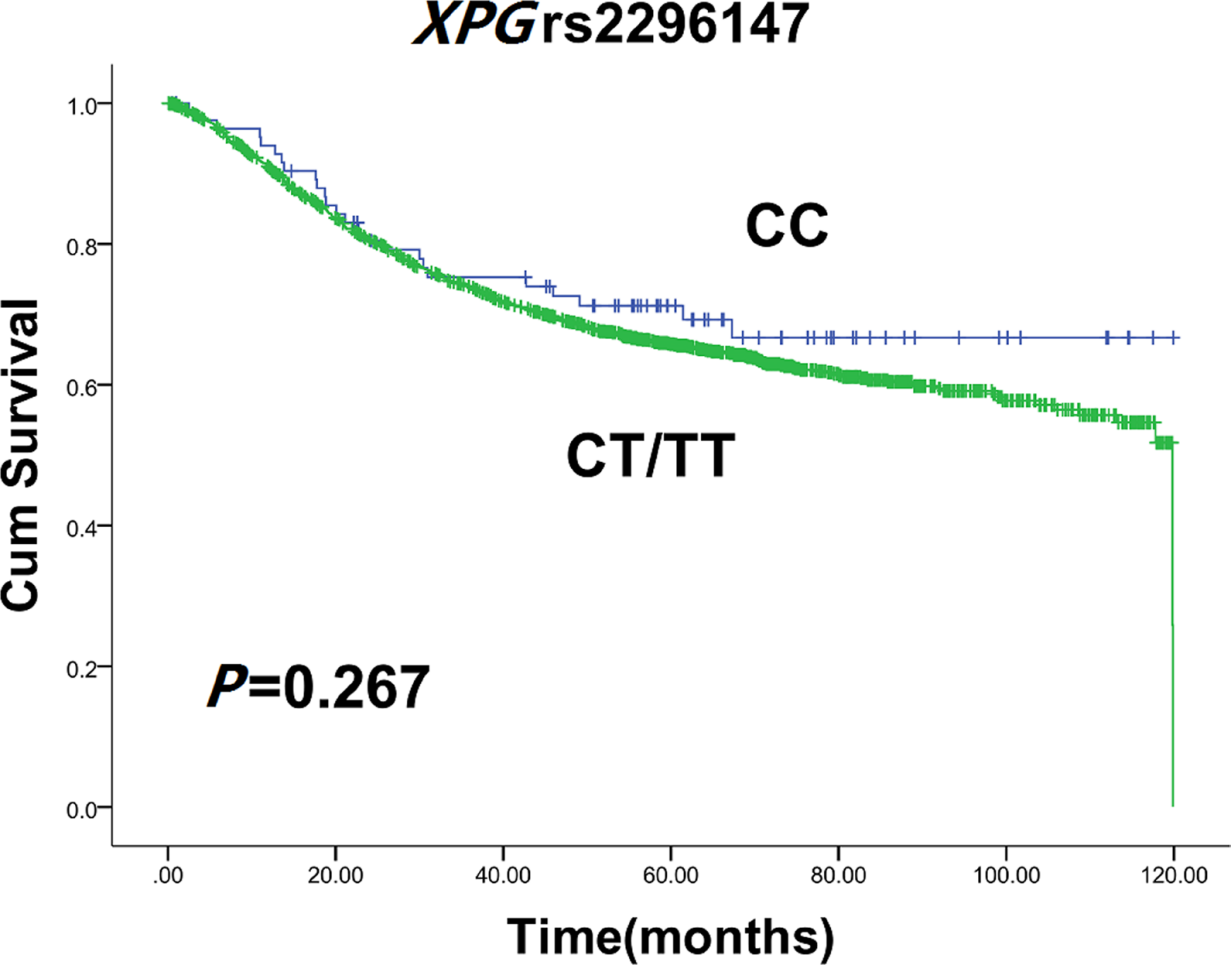
Kaplan-Meier estimates of 10 years OS with *XPG* rs2296147 CT/TT and CC genotypes in CRC patients

Genotype frequency distributions of four *XPG* SNPs were summarized in Table 2. The SNPs were analyzed for association with PFS and OS in patients with CRC. A significant association was found between *XPG* rs2296147 variant genotypes and PFS in CRC. *XPG* rs2296147 T>C polymorphism led to a decrease in median 10-year PFS time of 88.5 months for carriers of rs2296147 CT/TT genotype, when compared with 118.1 months for patients with CC genotype (log-rank test, *P* = 0.020) (Figure 1). Univariate Cox proportional hazards model analysis indicated that age, BMI, dukes stage, recurrence/metastasis status, and therapeutic response could influence 10 years OS or PFS (Table 3). After adjusting for those potential confounding factors, multivariate Cox proportional hazards model showed that patients carrying rs2296147 CT/TT genotype had an the hazard ratios (HR) of 1.324 (95% CI = 1.046–1.667) for developing progression in comparison non-carriers. It suggested that *XPG* rs2296147 CT/TT genotype might be an independent predictor of poor prognosis in CRC (Table 4). No association with PFS was observed for other SNPs (rs2094258, rs751402 and rs873601). Moreover, we failed to find any significant association for the *XPG* rs2296147 with 10-year OS (Figure 2), as well as the other three polymorphisms.

**Table 2:** Kaplan-Meier method and Cox proportional hazards model analysis of associations between the genotypes of *XPG* and CRC prognosis

Variants	10 years OS					10 years PFS				
	event	Median (months)	Log-rank *P*	HR (95% CI)	*P* ^a^	event	Median (months)	Log-rank *P*	HR (95%CI)	*P ^b^*
rs2094258										
TT	83	106.067		1.000		76	87.667		1.000	
CT	254	21.267	0.357	0.973 (0.747, 1.268)	0.842	208	98.633	0.169	0.85 (0.655, 1.108)	0.232
CC	265	119.800	0.945	0.942 (0.724, 1.223)	0.649	227	83.967	0.728	1.019 (0.785, 1.323)	0.889
CT/CC	519	119.800	0.600	0.978 (0.865, 1.106)	0.722	435	92.033	0.363	0.964 (0.854, 1.090)	0.561
rs2296147										
CC	25	20.067		1.000		18	118.067		1.000	
CT	200	119.800	0.392	1.381 (0.837, 2.276)	0.206	178	84.867	**0.024**	**1.780 (1.093, 2.898)**	**0.021**
TT	377	119.867	0.258	1.492 (0.915, 2.433)	0.108	315	89.567	**0.025**	**1.740 (1.080, 2.803)**	**0.023**
CT/TT	577	119.800	0.267	1.205(0.946,1.535)	0.131	493	88.500	**0.020**	**1.324 (1.046, 1.667)**	**0.020**
rs751402										
AA	83	119.867		1.000		76	83.867		1.000	
AG	263	119.800	0.489	0.953 (0.733, 1.240)	0.721	216	89.133	0.384	0.851 (0.655, 1.107)	0.851
GG	256	117.800	0.567	0.893 (0.688, 1.161)	0.399	219	92.033	0.521	0.830 (0.639, 1.080)	0.830
AG/GG	519	119.800	0.478	0.960 (0.850, 1.085)	0.515	435	91.233	0.414	0.917 (0.811, 1.036)	0.165
rs873601										
AA	155	117.800		1.000		144	85.300		1.000	
AG	286	119.800	0.347	1.028 (0,831, 1.272)	0.799	227	92.900	0.119	0.856 (0.694, 1.056)	0.856
GG	161	119.867	0.822	0.994 (0.780, 1.267)	0.964	140	93.267	0.502	0.969 (0.766, 1.226)	0.969
AG/GG	447	119.800	0.574	1.008 (0.912, 1.113)	0.877	367	93.267	0.162	0.946 (0.859, 1.043)	0.265

CRC, colorectal cancer; HR, hazard ratio; CI, confidence interval; OS, overall survival; PFS, progression-free survival.

a *P* values were calculated after adjustment for BMI, dukes stage, recurrence/metastasis status and therapeutic response.

b *P* values were calculated after adjustment for age and dukes stage.

**Table 3:** Cox proportional hazards model analysis of associations between demographic and clinical data of patients and CRC prognosis

Variables	*P*	HR	95% CI	
			Lower	Upper
10 years OS				
BMI	0.001	0.956	0.932	0.982
Therapeutic response	0.000	2.835	2.509	3.203
Dukes stage	0.000	3.613	3.226	4.046
Recurrence/Metastasis status	0.000	4.710	3.993	5.556
10 years PFS				
Age	0.002	0.990	0.984	0.996
Dukes stage	0.000	2.016	1.817	2.238

CRC, colorectal cancer; HR, hazard ratio; CI, confidence interval; OS, overall survival; BMI, body mass index; PFS, progression-free survival.

**Table 4:** Kruskal-Wallis analysis of associations between the demographic and clinical data of patients and genotypes of *XPG*

Variables	rs2094258		rs2296147		rs751402		rs873601	
	*P*^a^	*P*^b^	*P*^a^	*P*^b^	*P*^1^	*P*^2^	*P*^1^	*P*^2^
Sex	0.693	0.399	0.721	0.231	0.799	0.313	0.172	0.122
Age	0.107	0.026	0.666	0.544	0.121	0.054	0.063	0.106
Smoking status	0.509	0.537	0.576	0.539	0.410	0.898	0.929	0.800
Drinking status	0.299	0.510	0.170	0.271	0.282	0.713	0.671	0.789
BMI	0.682	0.764	0.612	0.812	0.523	0.615	0.830	0.919
Therapeutic response	0.538	0.865	0.230	0.181	0.232	0.550	0.824	0.963
Tumor site	0.569	0.241	0.708	0.408	0.751	0.818	0.796	0.447
Dukes stage	0.346	0.309	0.957	0.834	0.699	0.442	0.637	0.590

BMI, body mass index.

a *P* values were calculated for 10 years OS.

b *P* values were calculated for 10 years PFS.

## DISCUSSION

In the present study, we found that *XPG* rs2296147 CT/TT genotypes were correlated with poor 10-year PFS in CRC when compared with CC genotype. In other words, *XPG* rs2296147 CC genotype was associated with favorable prognosis regarding 10- year PFS, suggesting that *XPG* rs2296147 CC homozygous variation is a protective factor for the 10 years PFS in CRC. However, incidence of this homozygous variation is relatively low. There was no significant association between the rest of polymorphisms and clinical outcome of CRC patients.

*XPG* gene is mapped to chromosome 13q33, encoding an 1186 amino acid structure-specific endonuclease. During the process of NER, XPG is involved in the incision on 5′ side of damage and the maintenance of stability of TFIIH. Up to now, although an increasing number of studies focus on the common genetic variations in the NER pathway, studies on the association of *XPG* SNPs and the clinical outcome of CRC patients were limited. To the best of our knowledge, our study was the first large-scale case study to investigate the relationship between *XPG* polymorphisms of and prognosis of CRC. The *XPG* polymorphisms have been reported to affect the platinum-based chemotherapy sensitivity and prognosis of various cancers, including gastric cancer [18] and NSCLC [22]. Zhou et al. [19] found *XPG* rs2296147 and rs2094258 polymorphisms were associated with PFS and OS in NSCLC. Zhang et al. [23] also reported that down-regulation of XPG activity caused by rs2296147 polymorphism was correlated with increased OS. There are a number of studies evaluating the influence of the *XPG* SNPs on the risk and therapeutic response of CRC [15, 24–26]; however few have explored the association of *XPG* SNPs with the prognosis of CRC patients. CRC is one of the most common cancer worldwide, with approximately 55% of the cases occurring in the developed regions. So far, very few CRC-related studies involve Chinese populations [27, 28]. The current study might be the largest one that investigated the association of interest solely in Chinese by far. The association of polymorphisms in the NER pathway with CRC remains inconclusive. Moreno et al. [14] carried out a case-control study to assess gene-environment interactions by genotyping 28 SNPs in the 15 DNA repair genes among 377 CRC patients and 329 controls. Their results highlighted the important influence of SNPs in the DNA repair genes on the response to chemotherapy and prognosis of CRC patients. Meanwhile, Du et al. [25] found that *XPG* Asp1104His polymorphism was associated with a significantly increased risk of CRC, especially in Asian populations. However, Mort et al. [29] investigated polymorphisms in the NER genes (*XPD*, *XPF*, *XPG*, *ERCC1*) and failed to prove the important role of studied SNPs in protection against CRC. Zhu et al. [30] performed a meta-analysis to explore the relationship between the *ERCC5/XPG* Asp1104His polymorphism and cancer risk under the recessive genetic model, and found null association between the polymorphism and the risk of CRC. In contrast, our large-scale study provided evidence of the robust association between the *XPG* SNPs and the prognosis of CRC patients.

The *XPG* rs2296147 polymorphism is located in the 5′ untranslated regions (UTR), which was predicted to influence activity of transcription factor binding sites (TFBS) [31]. Cartharius et al. [32] found that *XPG* rs2296147 is a putative P53 transcription factor-binding site. Blomquist et al. [33] found that *XPG* rs2296147 is associated with altered allele-specific expression of XPG transcript in normal human bronchial epithelium, so the rs2296147 polymorphism may be important to XPG expression. Our study is the first one to validate the association between *XPG* rs2296147 polymorphism and risk of CRC, previous studies focus on how this site act on the clinical outcome of platinum-based chemotherapy in NSCLC patients [19, 22, 23, 34, 35], susceptibility of prostate cancer patients [36, 37], gastric cancer [38, 39], and breast cancer [20], but the number was limited. Our study is the first one to clarify the relationship between rs2296147 polymorphism and CRC survival.

In conclusion, our results indicated that *XPG* rs2296147 CT/TT was correlated with the prognosis of CRC patients. *XPG* rs2296147 polymorphism could be used as an independent predictive marker for the prognosis in CRC. Our study identified prognostic value of *XPG* SNPs. It might also serve as a molecular biomarker in individualized treatmen of colorectal cancer in future.

## MATERIALS AND METHODS

### Subject

A total of 1901 patients with histologically confirmed colorectal carcinoma were recruited from Sun Yat-Sen University Cancer Center between January 2000 to May 2010. We recruited patients without restrictions on age, sex, ethnicity, or clinical stage. We gained demographic and clinical data from medical record review, including age, sex, smoking status, drinking status, tumor site, therapeutic response, recurrence/metastasis status, and Dukes stage. Individuals who smoked cigarettes less than 1 package were defined as never smokers, while the others were ever smokers. An individual who drank alcohol less than 50 ml was defined as a never drinker, while the others were ever drinkers. All the patients were followed-up every year by telephone and deaths were recorded and confirmed by local Public Security Bureau until April 2015. All patients with informed consent donated their blood sample for the study.

### SNP selected and genotyping

Genomic DNA was obtained from the buffy coat fraction of each blood sample using a Qiagen Blood DNA Mini Kit (Qiagen Inc., Valencia, CA) according to the manufacturer's instructions. The candidate *XPG* SNPs were selected as reported previously [40]. Finally, four SNPs (rs2296147, rs2094258, rs751402, rs873601) in the *XPG* gene were chosen and included in the analysis. All these four selected SNPs were genotyped by using TaqMan real-time PCR as described previously [40]. For quality control, approximately 10% of the samples were randomly selected and repeatedly genotyped, and the results confirmed 100% concordance.

### Statistical methods

OS was defined as the time from the date of pathologically confirmed to the date of death or last clinical follow-up. PFS was calculated from the date of the pathologically confirmed to the progression of the disease, death without progression, or last clinical follow-up. Numerical variable in this study were expressed as mean and percentage. Survival distributions were estimated by using the Kaplan-Meier method and difference in the survival was tested using the log-rank test. To estimate the association of the four *XPG* SNPs with PFS and OS in CRC, the HR and 95% CI were calculated by univariate Cox proportional hazards model. Multivariate Cox model were performed to compute adjusted HR and 95% CI, after adjusting for potential risk factors. Homozygous variant genotype severed as a reference group. All tests were two-sided and *P* < 0.05 was considered to be significant. All statistics were conducted by SPSS 19.0 software.
